# Conditioning intensity in myelodysplastic patients aged ≥ 50 years undergoing allogeneic hematopoietic cell transplantation (allo-HCT): a study on behalf of the chronic malignancies working party of the EBMT

**DOI:** 10.1038/s41409-025-02682-3

**Published:** 2025-08-29

**Authors:** Noureddine Henoun Loukili, Luuk Gras, Linda Koster, Didier Blaise, Tobias Gedde-Dahl, Johan Maertens, Lone Smidstrup Friis, Stephan Mielke, Patrice Chevallier, Jakob R. Passweg, Jennifer Louise Byrne, Urpu Salmenniemi, Patrice Ceballos, Jérôme Cornillon, Simona Sica, Francesco Onida, Christof Scheid, Carmelo Gurnari, Joanna Drozd-Sokolowska, Kavita Raj, Marie Robin, Donal P. McLornan, Ibrahim Yakoub-Agha

**Affiliations:** 1 CHU de Lille, Lille, France; 2 EBMT Leiden Study Unit, Leiden, the Netherlands; 3 Institut Paoli Calmettes, Marseille, France; 4 Oslo University Hospital, Oslo, Norway; 5 UZ Gasthuisberg, Leuven, Belgium; 6 University Hospital, Rigshospitalet, and Institute of Clinical Medicine, University of Copenhagen, Copenhagen, Denmark; 7 Department of Cellular Therapy and Allogeneic Stem Cell Transplantation, Karolinska Institute & University Hospital, Stockholm, Stockholms Laen Sweden; 8 CHU de NANTES, Nantes Cedex 1, Nantes, France; 9 University Hospital, Basel, Switzerland; 10 Department of Hematology University Hospital of Basel, Basel, Switzerland; 11 Nottingham University, Nottingham, UK; 12 Department of Hematology, Helsinki University Hospital Comprehensive Cancer Center, Helsinki, Finland; 13 CHU Montpellier, Montpellier, France; 14 Institut de Cancerologie Lucien Neuwirth, CHU Saint Etienne, Saint Etienne, France; 15 Sezione di Ematologia, Dipartimento di Scienze Radiologiche ed Ematologiche, Università Cattolica del Sacro Cuore, Rome, Italy; 16 Universita Cattolica Sacro Cuore, Rome, Italy; 17 Fondazione IRCCS Cà Granda Ospedale Maggiore Policlinico - University of, Milan, Italy; 18 University of Cologne, Koeln, Germany; 19 Department of Translational Hematology and Oncology Research, Taussig Cancer Institute, Cleveland Clinic, Cleveland, OH &Department of Biomedicine and Prevention, University of Rome Tor Vergata, Rome, Italy; 20 Central Clinical Hospital, The Medical University of Warsaw, Warsaw, Poland; 21 University College London Hospitals NHS Trust, London, UK; 22 Hôpital Saint-Louis, APHP, Université de Paris Cité, Paris, France; 23 CHU de Lille, Univ Lille, INSERM U1286, Infinite, 59000 Lille, France

**Keywords:** Risk factors, Epidemiology

## Abstract

Reduced intensity conditioning (RIC) is usually used for patients with myelodysplastic syndrome (MDS) undergoing allogeneic hematopoietic cell transplantation (allo-HCT), particularly in the elderly or those with comorbidities. The impact of conditioning intensity on patients’ outcome remains controversial with clinicians’ subjective opinion/ experience remaining a major guide in choosing the intensity. Here, we compare RIC *versus* MAC in a large EBMT retrospective study in MDS patients aged ≥50 years undergoing allo-HCT between 2014 and 2018. Among the 1393 included patients, 922 (66%) were males, and the median age at transplant was 62.8 (50.0–77.9) years. The majority of patients (*n* = 884; 64.3%) had MDS with excess blasts. IPSS-R recorded was very low/low (*n* = 598, 43%), intermediate (*n* = 352, 25%), and high/very high (*n* = 443, 32%). Karnofsky index was ≥90 in 916 (69.3%) patients, and HCT-CI ≥ 3 in 292(27.3%) patients. A RIC regimen was used in 1053 (75.5%) patients. Median follow-up was 27.9 months (IQR: 26.4–30.6). Both uni- and multi-variable analyses did not show any significant association between conditioning intensity and outcomes. This study highlights a lack of association between RIC/MAC regimens and outcomes in allo-HCT MDS patients. Our results support the recently published systematic review and meta-analysis, where evidence for using one conditioning regimen over another remains weak.

## Introduction

Myelodysplastic syndromes/neoplasms (MDS) represent a heterogeneous group of clonal hematopoietic cell disorders with abnormal cellular maturation that results in ineffective hematopoiesis. This leads to variable degrees of cytopenias and an inherent, yet variable, risk of progression to acute myeloid leukemia (AML) [1]. Allogeneic hematopoietic-cell-transplantation (allo-HCT) remains the only treatment with curative potential, and is usually reserved for patients displaying high revised international prognostic scoring system (IPSS-R) risk scores [2, 3]. In the last two decades, reduced-intensity conditioning (RIC) regimens have emerged as an alternative to myeloablative conditioning (MAC) for allo-HCT preparation, particularly for the elderly or patients with high comorbidity scores, to limit the potential toxicity related to MAC platforms [4].

To date, most of the retrospective studies that have compared the two conditioning intensities have reported a higher risk of post-transplant relapse and a lower rate of non-relapse mortality (NRM) in the RIC groups. These studies included patients across a wide age range (18–70 years), albeit the median age of the patients in the RIC arms tended to be higher [5]. More recently, the results reported from the RIC/MAC Trial have provided evidence that utilization of RIC resulted in at least 2-years relapse-free survival and overall survival (OS) rates equivalent to MAC in MDS or secondary acute myeloid leukemia patients [6]. However, the median age of the included patients was 50 years, with a range of 19–65 years.

Studies addressing outcomes in RIC and MAC specifically in MDS patients are rare [7, 8]. Previous cohorts often included a mix of de novo AML and MDS patients, with a substantial proportion of AML and a broad age range. This heterogeneity makes it challenging to draw conclusions on the choice of the best type of conditioning tailored to the MDS patient population [9–12].

Being a universally established patient-specific variable crucial for outcome, age has been included in the HCT comorbidity index (HCT-CI) as a predictor of both NRM and OS [13, 14]. Patient’s age is indeed part of the consideration of the choice of conditioning regimen intensity, with the rational aim of reducing chemotherapy doses, potentially leading to less toxicity in older or frail patients. Given that MDS predominantly impacts older individuals, usually with a median age ranging from 70 to 75 years and only in rare instances allo-HCT is offered to those under 50 years old, our study specifically targeted MDS patients aged ≥ 50 years undergoing allo-HCT. This focus was intended to mitigate any potential impact that younger age might have on our study’s findings as well as to replicate the most common scenarios encountered in clinical practice.

With the initial intention to develop a score aiming to help the decision whether a given MDS patient can benefit from one conditioning intensity over the other, we conducted a comparison between RIC and MAC and studied the outcomes after allo-HCT in a large cohort of MDS patients aged ≥ 50 years at allo-HCT.

## Patients and methods

### Data source

This was a retrospective, multicenter, registry-based analysis approved by the Chronic Malignancies Working Party of the EBMT. The EBMT is a non-profit, scientific society representing more than 600 transplant centers mainly in Europe. Data are entered, managed, and maintained in a central database with internet access; each EBMT center is represented in this database. EBMT centers commit to obtain informed consent according to the local regulations applicable at the time of transplantation in order to report pseudonymized data to the EBMT. The study was approved by the EBMT-CMWP board and was conducted in accordance with the Declaration of Helsinki and Good Clinical Practice guidelines.

### Patient selection and outcome

MDS patients aged ≥50 years who received a first allo-HCT after RIC or MAC conditioning between 2014 and 2018 were selected from the database. Only patients for whom an IPSS-R score could be determined were included. Transplants with ex vivo T-cell depletion and myelodysplastic/myeloproliferative neoplasms (MDS/MPN) patients were excluded. RIC and MAC were classified as per established EBMT criteria [15] Outcomes studied were OS, EFS, relapse, and NRM. Overall survival (OS) was defined as the interval from allo-HCT to death, regardless of the cause of death. Event-free survival (EFS) was defined as survival with no evidence of relapse. Relapse was defined as the presence of more than 5% marrow blasts and/or reappearance of major myelodysplastic features associated with cytopenia (or worsening of previous cytopenia) EFS and evidence of autologous reconstitution when chimerism was available. Non-relapse mortality (NRM) was defined as death without evidence of relapse.

### Statistical methods

Baseline was defined as the day of the allo-HCT. For continuous baseline variables, results for RIC and MAC patients were expressed as medians and range/inter-quartile ranges (IQR). Categorical variables were described using frequencies and percentages. Multiple imputation, using the Mice package (3.16.0) [16]. In R, was then performed for variables with less than 40% missing data; otherwise, the variable was not included in multivariate analyses. Median follow-up after baseline and IQR were calculated using the reverse Kaplan-Meier (KM) method. OS and EFS were estimated using the Kaplan-Meier method, and the Log-Rank test was used to compare groups. Cumulative incidence rates (CIR) of relapse and NRM, considered as competing risks, were estimated using the crude cumulative incidence estimator and Gray’s test was used for comparing groups. Variables with a P value of less than 0.15 in univariable analyses were entered into a multivariate Cox proportional hazards (PH) model. Cause-specific hazards models were used for the analysis of relapse and NRM. The conditioning regimen was always included in the model, regardless of its significance level in the univariable analysis. Adjusted hazard ratios (aHRs) and their 95% confidence intervals (95% CIs) were estimated, and the threshold for statistical significance was set at *P* < 0.05. The PH assumption was tested using graphical diagnostics and statistical tests based on scaled Schoenfeld residuals. In case of violation of the proportionality of the hazard assumption, a stratified Cox proportional hazards model was performed. A propensity score matching (PSM) analysis [17] was also performed to adjust for variables that could potentially influence the outcome of allo-HCT and were associated with the use of RIC or MAC regimens. Therefore, multivariate logistic regression was performed to identify factors associated with conditioning regimen. Factors with a *p*-value < 0.05 were then included in the matching process. Patients were propensity score matched 1:1 without replacement using the nearest neighbor method and a caliper of 0.1. The balance between the matched groups was checked using the mean standardized differences between the two groups. As the matched patients do not consist of independent observations, a conditional logistic regression model was used to estimate the association between RIC/MAC regimen and outcomes. In case of the lack of association between RIC/MAC conditioning regimen and outcomes, a post-hoc analysis was performed to identify potential subgroups that would benefit from RIC or MAC conditioning regimen. Finally, we applied the subgroup identification model, using machine learning, proposed by Huling et al. [18]. and applied by Shimomura et al. for identification of subgroups of MDS patients for whom RIC or MAC could be the optimal conditioning regimen for allo-HCT [19]. This method uses a propensity score weighted model to estimate interactions between covariates and conditioning regimens, incorporating eighteen covariates detailed in the supplementary materials. Lasso regularization was employed to select key covariates for subgroup identification. The “benefit score (BS) for RIC,” was calculated by summing the interaction terms between the treatment and covariates. Individuals were divided into two groups based on the first quartile of BS. Supervised learning techniques were then used to explore how covariates relate to outcomes, considering the RIC regimen’s effect. The treatment effect for each person was estimated by comparing their predicted outcomes with and without the treatment, identifying subgroups with similar treatment effects and profiles. This process was carried out using the “personalized” package in R [18].

All statistical analyses were performed using R.3.6.3 software (R Foundation for Statistical Computing, Vienna, Austria. Strengthening the Reporting of Observational studies in Epidemiology (STROBE) criteria were used to report the study results [20].

## Results

### Patient characteristics

A total of 1393 MDS patients who underwent a first allo-HCT during 2014-2018 were enrolled in the study. Of these, 1053 (75%) received a RIC regimen, while the remaining 340 (25%) patients received a MAC regimen. Overall, median follow-up was 27.9 months (95%CI: 26.4–30.6). The male:female ratio was 1.95 and the median age of the entire cohort was 62.8 years (range: 50.0–77.9). The cytogenetic IPSS-R score at transplant was stratified as lower risk (very low/low) (*n* = 25/907, 65.1%), intermediate (*n* = 250, 17.9%) and higher risk (poor/very poor) (*n* = 111/100, 8%/7.2%). Most patients had a Karnofsky performance score (KPS) ≥ 90 (*n* = 916, 65.8%), and a Hematopoietic Cell Transplantation-specific Comorbidity Index (HCT-CI) ≥ 1 (*n* = 588, 42.2%). Disease status at transplant was CR in approximately one-third of evaluable cases (*n* = 486, 34.9%) while 252 patients (18.1%) did not receive pre-transplant treatment. Donors consisted of HLA-matched related/unrelated (*n* = 989, 71%), HLA-mismatched unrelated (*n* = 250, 17.9%) and haploidentical (*n* = 153, 11%). Table 1 depicts disease features, patients’ and donors’ characteristics as well as transplantation modalities.

**Table 1:** Disease features, patients’ and donors’ characteristics and transplantation modalities.

	Overall (*N* = 1393)	MAC group (*N* = 340)	RIC group (*N* = 1053)
Age, median (range), years	62.8 (50.0, 77.9)	59.7 (50.0, 75.4)	63.7 (50.0, 77.9)
Patient’s sex ratio (M/F)	1.95	1.63	2.07
Year of transplant			
2014	281 (20.2%)	57 (16.8%)	224 (21.3%)
2015	279 (20.0%)	68 (20.0%)	211 (20.0%)
2016	250 (17.9%)	64 (18.8%)	186 (17.7%)
2017	273 (19.6%)	70 (20.6%)	203 (19.3%)
2018	310 (22.3%)	81 (23.8%)	229 (21.7%)
WHO classification at transplant, *n* (%)			
del5q	16 (1.1%)	5 (1.5%)	11 (1.0%)
MDS Unclassifiable/other	100 (7.2%)	26 (7.6%)	74 (7.0%)
RA without ring sideroblasts	21 (1.5%)	2 (0.6%)	19 (1.8%)
RAEB	0 (0%)	0 (0%)	0 (0%)
RAEB-1	294 (21.1%)	72 (21.2%)	222 (21.1%)
RAEB-2	590 (42.4%)	152 (44.7%)	438 (41.6%)
RARS (with ring sideroblasts)	32 (2.3%)	5 (1.5%)	27 (2.6%)
RCMD	130 (9.3%)	32 (9.4%)	98 (9.3%)
RCMD-RS	32 (2.3%)	5 (1.5%)	27 (2.6%)
Transformed to AML	161 (11.6%)	36 (10.6%)	125 (11.9%)
missing	17 (1.2%)	5 (1.5%)	12 (1.1%)
White blood cells 10^9^/L, median (Range)	2.90 (0.200, 5980)	2.90 (0.200, 5100)	2.90 (0.500, 5980)
missing	50 (21.6%)	157 (20.9%)	207 (21.1%)
Neutrophils 10^9^/L, median (range)	1.07 (0, 14.6)	0.850 (0, 14.6)	1.13 (0.07, 12.5)
missing	902 (91.8%)	207 (89.2%)	695 (92.5%)
Peripheral blast count, median (Range)	0 (0, 70.0)	0 (0, 70.0)	0 (0, 27.0)
missing	421 (42.8%)	82 (35.3%)	339 (45.1%)
Marrow blast count, median (Range)	8.00 (0, 95.0)	7.00 (0, 72.0)	8.00 (0, 95.0)
missing	156 (15.9%)	34 (14.7%)	122 (16.2%)
Hemoglobin g/dL, median (Range)	9.90 (1.40, 30.0)	10.2 (3.50, 30.0)	9.90 (1.40, 16.4)
missing	180 (18.3%)	42 (18.1%)	138 (18.4%)
Cytogenetic IPSS-R, *n* (%)			
Very good	25 (1.8%)	4 (1.2%)	21 (2.0%)
Good	907 (65.1%)	223 (65.6%)	684 (65.0%)
Intermediate	250 (17.9%)	68 (20.0%)	182 (17.3%)
Poor	111 (8.0%)	21 (6.2%)	90 (8.5%)
Very poor	100 (7.2%)	24 (7.1%)	76 (7.2%)
IPSS-R, *n* (%)			
Very low	215 (15.4%)	44 (12.9%)	171 (16.2%)
Low	383 (27.5%)	91 (26.8%)	292 (27.7%)
Intermediate	352 (25.3%)	86 (25.3%)	266 (25.3%)
High	265 (19.0%)	72 (21.2%)	193 (18.3%)
Very high	178 (12.8%)	47 (13.8%)	131 (12.4%)
Karnofsky index, *n* (%)			
≥90	916 (65.8%)	244 (71.8%)	672 (63.8%)
<90	404 (29.0%)	82 (24.1%)	322 (30.6%)
missing	73 (5.2%)	14 (4.1%)	59 (5.6%)
Disease status, *n* (%)			
Complete remission	486 (34.9%)	98 (28.8%)	388 (36.8%)
Partial remission	21 (1.5%)	7 (2.1%)	14 (1.3%)
Stable disease/Untreated	535 (38.4%)	153 (45.0%)	382 (36.3%)
Relapse/progression/refractory	310 (22.3%)	75 (22.1%)	235 (22.3%)
Other	19 (1.4%)	3 (0.9%)	16 (1.5%)
missing	22 (1.6%)	4 (1.2%)	18 (1.7%)
HCT-CI risk, *n* (%)			
low risk (0)	479 (34.4%)	123 (36.2%)	356 (33.8%)
intermediate risk (1–2)	296 (21.2%)	74 (21.8%)	222 (21.1%)
high risk (≥3)	292 (21.0%)	79 (23.2%)	213 (20.2%)
missing	326 (23.4%)	64 (18.8%)	262 (24.9%)
CMV serostatus recipient/donor, *n* (%)			
−/−	403 (28.9%)	93 (27.4%)	310 (29.4%)
−/+	115 (8.3%)	25 (7.4%)	90 (8.5%)
+/−	367 (26.3%)	98 (28.8%)	269 (25.5%)
+/+	482 (34.6%)	119 (35.0%)	363 (34.5%)
missing	26 (1.9%)	5 (1.5%)	21 (2.0%)
Donor type, *n* (%)			
matched related (MRD)	315 (22.6%)	77 (22.6%)	238 (22.6%)
Haplo	153 (11.0%)	36 (10.6%)	117 (11.1%)
matched unrelated (MUD)	674 (48.4%)	164 (48.2%)	510 (48.4%)
mismatched unrelated (MMUD)	194 (13.9%)	41 (12.1%)	153 (14.5%)
unrelated	56 (4.0%)	22 (6.5%)	34 (3.2%)
missing	1 (0.1%)	0 (0%)	1 (0.1%)
In vivo T-cell depletion, *n* (%)			
no	452 (30.2%)	122 (35.8%)	330 (31.3%)
Mab-campath	60 (4.3%)	3 (0.8)	57 (5.4%)
Anti-thymoglobulin	881 (63.2%)	215 (63.2)	666 (63.3)
Total body irradiation, *n* (%)			
no	1201 (86.2%)	328 (96.5%)	873 (82.9%)
yes	186 (13.4%)	10 (2.9%)	176 (16.7%)
missing	6 (0.4%)	2 (0.6%)	4 (0.4%)
Prior to transplant lines of therapy, *n* (%)			
0	252 (18.1%)	74 (21.8%)	178 (16.9%)
1	961 (69.0%)	211 (62.1%)	750 (71.2%)
>1	104 (7.4)	30 (8,8)	67 (6,3)
missing	73 (5.2%)	25 (7.4%)	48 (4.6%)
Prior to transplant HMA			
No	757 (54.3%)	170 (50.0%)	587 (55.7%)
Yes	554 (39.8%)	143 (42.1%)	411 (39.0%)
Missing	82 (5.9%)	27 (7.9%)	55 (5.2%)

*HCT-CI* hematopoietic cell transplantation-comorbidity index, *HMA* hypomethylating agents

Values are kept at transplant.

### Survival outcomes

**Fig. 1:**
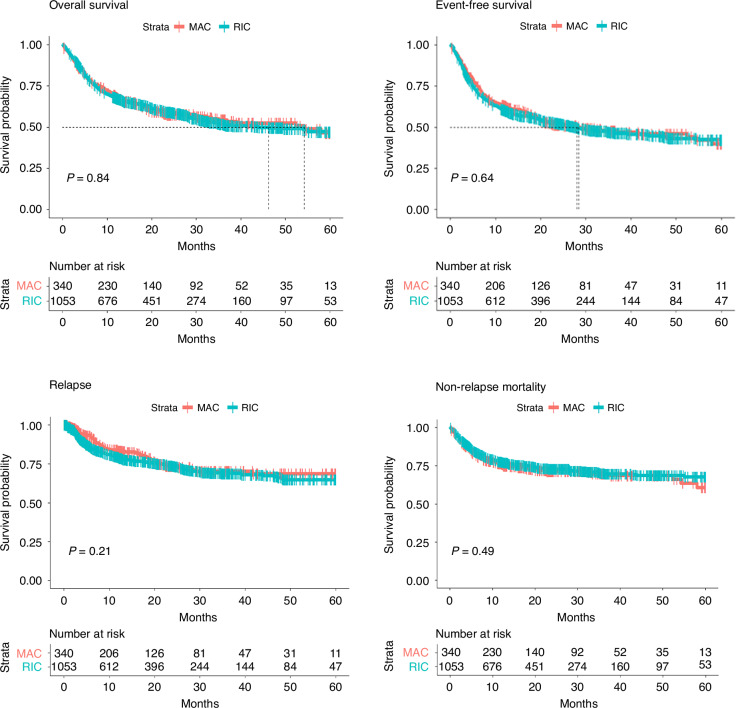
Kaplan-Meier curves and Log-rank of the association of RIC/MAC conditioning regimens on Overall survival, Event-free survival, Relapse and Non-relapse mortality outcomes.

**Fig. 2:**
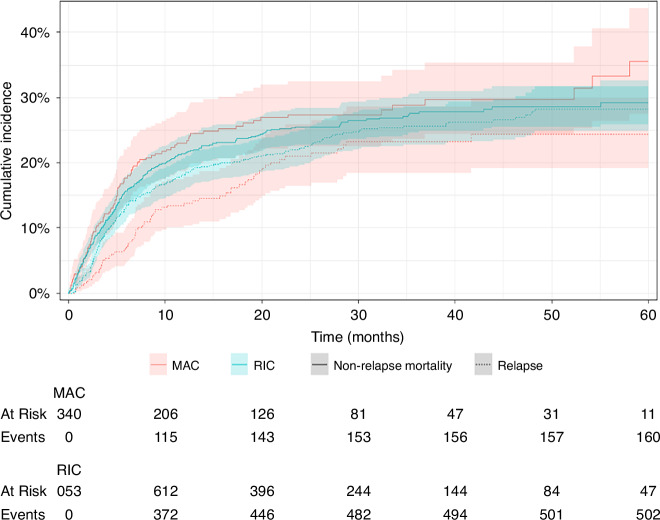
Cumulative incidences of relapse and non-relapse mortality depending on the intensity of the conditioning regimen (RIC/MAC). RIC reduced intensity conditioning, MAC myeloablative conditioning.

In the analysis of the entire cohort, the estimated 3-year OS rates were 51.8% (95% CI: 48.3–55.5) for the RIC group and 54.4% (95% CI: 48.6–60.8) for the MAC group. Similarly, the estimated 3-year EFS rates were 46.7% (95% CI: 44.4–50.3) for RIC and 47.9% (95% CI: 42.1–54.4) for MAC (Fig. 1). No significant differences were observed in the 3-year CIR of relapse or NRM between the RIC and MAC regimens (Gray’s test, *p* = 0.17 and *p* = 0.38, respectively). The 3-year CIR of relapse was 23.2% (95% CI: 22.7–28.6) for RIC and 25.6% (95% CI: 18.3–28.5) for MAC. For 3-year NRM, the rates were 28.8% (95% CI: 24.6–30.6) for RIC and 27.6% (95% CI: 23.6–34.1) for MAC (Fig. 2).

### Outcome predictors and conditioning intensity

Univariable analyses revealed several significant associations with OS. Specifically, age at allo-HCT over 60 years [HR = 1.25, 95%CI: 1.05-1.49, *p* = 0.014], KPS < 90 [HR = 1.19, 95%CI: 1.00–1.42, *p* = 0.045], HCT-CI ≥ 3 [HR = 1.24, 95%CI: 1.02–1.50, *p* = 0.03], and patient seropositivity for CMV [HR = 1.26, 95%CI: 1.06–1.49, *p* = 0.009] were associated with shorter survival. Additionally, the transformation into AML [HR = 1.46, 95%CI: 1.18–1.81, *p* = 0.001], blasts in peripheral blood >1% [HR = 2.16, 95%CI: 1.82–2.56, *p* < 0.001], and hemoglobin <10 g/dL at transplant [HR = 1.31, 95%CI: 1.11–1.55, *p* = 0.001], poor/very poor cytogenetic IPSS-R score [HR = 1.72, 95%CI: 1.40–2.11, *p* < 0.001], and use of an HLA mismatched donor (related or unrelated) [HR = 1.38, 95%CI: 1.16–1.64, *p* < 0.001] were also significantly associated with shorter survival. However, the intensity of conditioning regimen did not show any significant association with shorter OS [HR = 1.02, 95%CI: 0.84–1.23, *p* = 0.83].

In multivariate analysis, RIC/MAC regimen use was not significantly associated with OS [HR = 1.14, 95%CI: 0.76–1.73, *p* = 0.52]. Only transformation into AML [HR = 1.34, 95%CI: 1.06–1.69, *p* = 0.015], high HCT-CI ≥ 3 [HR = 1.58, 95%CI: 1.07–2.33, *p* = 0.02], and the presence of blasts >1% in the peripheral blood [HR = 1.58, 95%CI: 1.07–2.33, *p* = 0.02] remained significant predictors of OS in the final model (Table 2).

**Table 2:** Univariate and multivariate analysis for overall survival according to patient’s, disease and transplantation characteristics.

	Level	All patients	Univariate analysis HR (CI95%, *p*-value)	Multivariate analysis HR (CI95%, *p*-value)
Age	<60	466 (33.5)	−	−
	>=60	927 (66.5)	1.25 (95% CI: 1.05–1.49, *p* = 0.014)	1.08 (95% CI: 0.78–1.51, *p* = 0.634)
Sex	Male	922 (66.2)	−	
	Female	471 (33.8)	0.94 (95% CI: 0.79–1.11, *p* = 0.457)	
Secondary AML	No	1233 (88.5)	−	−
	Yes	160 (11.5)	1.41 (95% CI: 1.12–1.77, *p* = 0.003)	1.34 (95% CI: 1.06–1.69, *p* = 0.015)
Cytogenetic IPPS-R	Very good/good	932 (66.9)	−	−
	Intermediate	250 (17.9)	1.26 (95% CI: 1.02–1.57, *p* = 0.033)	1.13 (95% CI: 0.74–1.74, *p* = 0.564)
	Poor/very poor	211 (15.1)	1.72 (95% CI: 1.40–2.11, *p* < 0.001)	1.02 (95% CI: 0.59–1.78, *p* = 0.935)
Karnofsky performance	>90	964 (69.2)	−	−
	<90	429 (30.8)	1.19 (95% CI: 1.00–1.42, *p* = 0.045)	1.00 (95% CI: 0.70–1.43, *p* = 0.993)
IPSS-R	Very low/low	598 (42.9)	−	
	Intermediate/high	617 (44.3)	1.21 (95% CI: 1.01–1.45, *p* = 0.040)	
	Very high	178 (12.8)	2.14 (95% CI: 1.70–2.69, *p* < 0.001)	
HCT-CI risk score	1	629 (45.2)	−	−
	2	369 (26.5)	1.16 (95% CI: 0.95–1.41, p = 0.158)	1.23 (95% CI: 0.81–1.88, *p* = 0.329)
	3	395 (28.4)	1.24 (95% CI: 1.02–1.50, *p* = 0.030)	1.58 (95% CI: 1.07–2.33, *p* = 0.021)
Recipient-CMV serostatus	Negative	530 (38.0)	−	−
	Positive	863 (62.0)	1.26 (95% CI: 1.06–1.49, *p* = 0.009)	1.30 (0.92–1.83, *p* = 0.136)
Donor type	MRD/MUD	990 (71.1)	−	−
	Haplo/MMUD	403 (28.9)	1.38 (1.16–1.64, *p* < 0.001)	1.32 (95% CI: 0.93–1.86, *p* = 0.119)
Source of graft	Peripheral blood	1254 (90.0)	−	
	Others	139 (10.0)	1.22 (95% CI: 0.94–1.58, *p* = 0.145)	
Reduced intensity conditioning	No	340 (24.4)	−	−
	Yes	1053 (75.6)	1.02 (95% CI: 0.84–1.23, *p* = 0.839)	1.14 (95% CI: 0.76–1.73, *p* = 0.526)
Prior to transplant HMA	No	597 (42.9)	−	−
	Yes	796 (57.1)	0.84 (95% CI: 0.71–0.99, *p* = 0.033)	0.86 (95% CI: 0.61–1.21, *p* = 0.386)
Total body irradiation	No	1206 (86.6)	−	
	*Yes*	187 (13.4)	0.95 (95% CI: 0.75–1.20, *p* = 0.655)	
Pre-treatment	No	1166 (83.7)	−	
	Yes	227 (16.3)	1.13 (95% CI: 0.91–1.40, *p* = 0.278)	
Peripheral blast count	0	1058 (76.0)	−	−
	1	335 (24.0)	2.16 (95% CI: 1.82–2.56, *p* < 0.001)	1.58 (95% CI: 1.07–2.33, *p* = 0.021)
Marrow blasts count	<=2	235 (56.4)	−	−
	>2	182 (43.6)	1.05 (95% CI: 0.77–1.44, *p* = 0.744)	0.96 (95% CI: 0.69–1.33, *p* = 0.800)
Platelet (10^9^/L)	>=100	192 (13.8)	−	−
	<100	1201 (86.2)	1.21 (95% CI: 0.94–1.56, *p* = 0.137)	0.99 (95% CI: 0.63–1.56, *p* = 0.968)
Disease status	Complete remission	490 (35.2)	−	
	Other status	903 (64.8)	1.11 (95% CI: 0.93–1.31, *p* = 0.255)	
Hemoglobin (g/dL)	≥10	647 (46.4)	−	−
	<10	746 (53.6)	1.31 (95% CI: 1.11–1.55, *p* = 0.001)	1.17 (95% CI: 0.84–1.63, *p* = 0.360)
Neutrophil count 10^9^/L	≥0.8	773 (55.5)	−	−
	<0.8	620 (44.5)	1.15 (95% CI: 0.98–1.36, *p* = 0.089)	0.73 (95% CI: 0.52–1.01, *p* = 0.059)
T-cell depletion	No	452 (32.4)	−	
	Yes	941 (67.6)	1.04 (95% CI: 0.87–1.24, *p* = 0.654)	

Similar results were observed in the univariable analyses of EFS, with all aforementioned covariates showing significant associations with EFS. Of note, there was no significant association between the intensity of conditioning regimen and EFS [HR = 1.04, 95%CI: 0.87–1.25, *p* = 0.64].

In multivariate analysis of EFS, the final model was stratified on IPSS-R cytogenetic score to comply with the proportional hazard assumption. The results showed that transformation into AML [HR = 1.39, 95%CI:1.12–1.73, *p* = 0.003], HCT-CI score ≥3 (HR = 1.33, 95%: 1.11–1.60, *p* = 0.002], patient seropositivity for CMV [HR = 1.24, 95%CI: 1.05–1.46, *p* = 0.009], HLA-mismatched donor (related and unrelated) [HR = 1.24, 95%CI: 1.05–1.46, *p* = 0.011] and the presence of >1% blasts in the peripheral blood [HR = 2.09, 95%: 1.77–2.47, *p* < 0.001] were associated with a shorter EFS (Supplementary Table 1s).

### Outcomes analysis after propensity score matching

Supplementary Table 2s presents the characteristics of patients after applying PSM. This PSM sample comprised 666 patients, evenly split between the two conditioning regimens (RIC and MAC), ensuring balanced pre-transplant variables between the groups (Supplementary Table 3s). A multivariate conditional logistic regression analysis indicated no significant impact of the conditioning regimen’s intensity on outcomes. Specifically, the results were as follows: OS [HR = 0.83, 95% CI: 0.61–1.14, *p* = 0.26], EFS [HR = 0.81, 95% CI: 0.59–1.10, *p* = 0.18], relapse/CIR [HR = 0.91, 95% CI: 0.64–1.29, *p* = 0.59], and NRM [HR = 1.11, 95% CI: 0.68–1.80, *p* = 0.68]. The outcomes for both the full cohort and the PSM analysis are detailed in Table 3.

**Table 3:** Impact of the intensity of the conditioning regimen on outcomes.

Full cohort analysis			Propensity score matching analysis	
	Univariate	Multivariate	Univariate	Multivariate
Overall survival	1.02 (95%CI: 0.84–1.23, *p* = 0.839)	1.14 (95%CI: 0.76–1.73, *p* = 0.526)	0.84 (95%CI: 0,62–1,85); p = 0,274)	0.83 (95%CI: 0.61–1.14, p = 0.264)
Progression free survival	1.04 (95%CI: 0.87–1.25, *p* = 0.636)	1.02 (95%CI: 0.85–1.23, *p* = 0.831)	0.81 (95%CI: 0.60–1.10, p = 0,188)	0.81(95%CI: 0.59–1.10, p = 0. 180)
Non-relapse mortality	0.97 (95%CI: 0,82–1.15, *p* = 0.802)	0.91 (95%CI: 0.76–1.09, *p* = 0.298)	0.83 (95%CI: 0.61–1.12, p = 0,228)	1.11 (95%CI: 0.68–1.80, p = 0.680)
Relapse	1.19 (95%CI: 0.86–1.56, *p* = 0.205)	1.18 (95%CI: 0.88–1.60, *p* = 0.273)	1.33 (95%CI: 0.92–1.91, p = 0.120)	0.91 (95%CI: 0.64–129, p = 0.598)

Hazard ratio for the full cohort and propensity score matching (PSM).

### Subgroup analysis

In the subgroup identification approach, only the interaction of the sex covariate with the conditioning regimen was selected by the lasso regression and included in the BS calculation for OS and NRM, respectively. This lead to identify two subgroups which could benefit from either RIC or MAC for each outcome. Nevertheless, no significant difference (*p*-value ≥ 0.1) between RIC and MAC regimens were registered for both OS and NRM for all identified subgroups (Supplementary Fig. 1s). No subgroup was identified for the relapse model.

### Sensitivity analysis

Excluding non-transformed AML patients from the overall cohort yields similar results to those observed in the full cohort for the outcomes considered. Therefore, the estimated 3-year OS rates were 53.5% (95% CI: 49.8–55.7) for the RIC group and 55.1% (95% CI: 49.0–60.9) for the MAC group. The estimated 3-year EFS rates were 46.7% (95% CI: 44.4–50.3) for RIC and 47.9% (95% CI: 42.1–54.4) for MAC (Supplementary Fig. 2s). No significant differences were observed in the 3-year CIR of relapse or NRM between the RIC and MAC regimens (Gray’s test, p = 0.28 and *p* = 0.39, respectively). The 3-year CIR of relapse was for 24.3% (95% CI: 21.3–27.5) RIC and 22.9% (95% CI: 17.7–28.5) for MAC. For 3-year NRM, the rates were 27.1% (95% CI: 24.0–30.0) for RIC and 28.9% (95% CI: 23.4–34.6) for MAC.

## Discussion

In this study, we investigated the influence of conditioning intensity on the outcomes of allo-HCT in patients with MDS aged ≥50 years. We specifically focused on this age group because it is well established that age independently affects outcomes of MDS patients undergoing allo-HCT, as reported in various literature sources, including the adapted EBMT score [21]. Our study differs from other research in its use of a homogeneous and “real-life” reflecting population of MDS patients. Thus, we intentionally excluded individuals with de novo AML, due to the substantial disparities in prognosis and pre-transplant management.

The primary finding of our analysis is the lack of a significant difference between MAC and RIC across all assessed outcomes, which encompassed OS, EFS, CIR, and NRM. EFS Despite our thorough examination to pinpoint specific subgroups that might derive advantage from either type of conditioning intensity, we could not identify such subgroups. Hence, creating a scoring system to aid clinicians in choosing the optimal conditioning regimen for MDS patients undergoing allo-HCT is challenging. These results are similar to those obtained after excluding patients with transformed AML from the initial cohort, underscoring that AML transformation does not influence the effect of MAC or RIC on patient outcomes.

Overall, our findings align with those reported in the existing literature, which also failed to identify a subgroup of patients benefitting from either RIC or MAC. In their systematic review and meta-analysis, Rashidi et al. compared the results of RIC and MAC allo-HCT in MDS patients [22]. They found no significant difference in overall outcomes between the two strategies. Nevertheless, this meta-analysis only included two prospective randomized studies, each with limited cohort sizes (54 and 129 patients, respectively). Thus, it is reasonable to infer that the study’s statistical power might not have allowed detection of variations in outcomes between RIC and MAC, especially in such a biologically and clinically heterogeneous disease as MDS. Additionally, the age range of the patients in these studies spanned from 19 to 66 years, while our analysis specifically focused on patients aged over 50 years, leveraging the larger sample size provided by the EBMT registry. We felt compelled to such an adjustment of selection criteria to be able to capture the most common clinical scenarios encountered in our daily clinical practice. In fact, median age of MDS at presentation lies in between the 7th and 8th decades of life [23].

Dillon et al. conducted a randomized phase III study to investigate the impact of conditioning intensity and genomics on post-allogeneic HCT relapse in MDS patients [24]. They observed that targeted DNA sequencing of 10 genes (most of which are more common in AML) before allo-HCT could identify MDS cases at highest risk of post-transplant relapse. Among patients who tested positive for such mutations, random assignment to MAC reduced the risk of relapse, resulting in a significantly better EFS than if they underwent a RIC transplant [24]. Unfortunately, we were unable to assess the impact of mutational status, as we lacked access to this data, which can further provide crucial prognostic information in the current molecular era, beyond or along with patient-specific factors such as age.

In another randomized study, Sharma et al. reported the long-term advantage of MAC conditioning over RIC. It is worth noting that the population in their study differed from ours, as it included again younger patients with AML and MDS with fewer than 5% blast cells in the marrow, along with HLA-compatible donors [11]. Similarly, the retrospective study conducted by Bejanyan et al. included younger patients, most of whom had AML, making it challenging to draw conclusive results for the MDS population [25]. However, the latter study, including also the consideration of the Disease Risk Index (DRI) that considers the cytogenetic risk and disease status at allo-HCT, did not observe higher CIR and lower NRM when using RIC regimens in high/very high DRI cases, while an increased EFS was noticed in the low/intermediate group [24]. A recent phase 3 trial comparing RIC and sequential regimen with fludarabine/amsacrine/cytarabine-busulphan (FLAMSA-Bu) has reported similar outcomes in both arms [26].

One limitation of our study is that we could only adjust our estimates for a standard set of variables, which, albeit most frequent, might not be the only ones used in the decision to offer RIC versus MAC. Another limitation is that subjective opinions and experiences of clinicians may significantly influence the selection of the appropriate conditioning intensity for their patients, potentially complicating retrospective studies and hindering the drawing of definitive conclusions. On the other hand, our study suffers the absence of mutational data, as these transplants occurred before the publication and wider spread use of the molecular IPSS score [27]. However, we provided detailed clinical information on a large sample size with adequate post allo-HCT follow-up.

In conclusion, this large cohort study did not underline any significant difference favoring one conditioning intensity over the other for patients with MDS aged ≥50 years undergoing allo-HCT. The integration of molecular profiling data in future studies may potentially aid identification of specific patient subgroups that may benefit from either RIC or MAC regimens.

## Supplementary information

Supplemental Material

## Data Availability

Questions regarding data sharing should be addressed to the corresponding author.
