# Supplementary material for: Conditioning intensity in myelodysplastic patients aged ≥ 50 years undergoing allogeneic hematopoietic cell transplantation (allo-HCT): a study on behalf of the chronic malignancies working party of the EBMT
Source: Bone Marrow Transplant. 2025 Aug 29;60(11):1487–95. doi: 10.1038/s41409-025-02682-3 (PMC12583188; doi:10.1038/s41409-025-02682-3)
Supplement: Supplementary file 1 — Supplemental Material [file 41409_2025_2682_MOESM1_ESM.docx]

**Supplementary appendix**

**Table 1s**: Univariate and multivariate analysis of Event-free Survival according to patient’s characteristics

|  |  | All patients | Univariate analysis  HR (95% CI, p-value) | Multivariate analysis  HR (95% CI, p-value) |
| --- | --- | --- | --- | --- |
|  |  |  |  |  |
| *Age* | <60 | 466 (33.5) | - | - |
|  | >=60 | 927 (66.5) | 1.22 (95% CI: 1.04-1.44, p=0.018) | 1.15 (95% CI: 0.97-1.37, p=0.109) |
| *Sexe* | Male | 922 (66.2) | - |  |
|  | Female | 471 (33.8) | 0.99 (95% CI: 0.84-1.16, p=0.897) |  |
| *WHO classification* | Others | 311 (22.3) | - |  |
|  | RAEB-1&2,RA & RCMD | 1082 (77.7) | 0.88 (95% CI: 0.74-1.05, p=0.169) |  |
| *Secondary AML* | no | 160 (11.5) | - | - |
|  | yes | 1233 (88.5) | 1.46 (95% CI: 1.18-1.81, p=0.001) | 1.39 (95% CI: 1.12-1.73, p=0.003) |
| *Cytogentic score* | vgood/good | 932 (66.9) | - |  |
|  | intermediate | 250 (17.9) | 1.21 (95% CI: 0.99-1.48, p=0.067) |  |
|  | poor/vpoor | 211 (15.1) | 1.74 (95% CI: 1.43-2.11, p<0.001) |  |
| *Kranofsky performance* | >=90 | 964 (69.2) | - |  |
|  | <90 | 429 (30.8) | 1.14 (95% CI: 0.97-1.34, p=0.110) |  |
| *IPSSR* | v.low/low | 598 (42.9) | - |  |
|  | intermediate/high | 617 (44.3) | 1.22 (95% CI: 1.03-1.44, p=0.022) |  |
|  | v. high | 178 (12.8) | 1.98 (95% CI: 1.59-2.46, p<0.001) |  |
| *HCT-CI risk* | 1 | 629 (45.2) | - | - |
|  | 2 | 369 (26.5) | 1.18 (95% CI: 0.98-1.42, p=0.087) | 1.14 (95% CI: 0.94-1.38, p=0.188) |
|  | 3 | 395 (28.4) | 1.29 (95% CI: 1.08-1.55, p=0.005) | 1.33 (95% CI: 1.11-1.60, p=0.002) |
| *CMV status of patients* | pat.cmv- | 530 (38.0) | - | - |
|  | pat.cmv+ | 863 (62.0) | 1.24 (95% CI: 1.06-1.46, p=0.008) | 1.24 (95% CI: 1.05-1.46, p=0.009) |
| *Donor type* | MRD/MUD | 990 (71.1) | - | - |
|  | Haplo/MMUD | 403 (28.9) | 1.28 (95% CI: 1.09-1.51, p=0.003) | 1.24 (95% CI: 1.05-1.46, p=0.011) |
| *Source of graft* | peripheral blood | 1254 (90.0) | - |  |
|  | others | 139 (10.0) | 1.16 95% CI: (0.91-1.49, p=0.226) |  |
| *Reduced intesity conditionning* | no | 340 (24.4) | - | - |
|  | yes | 1053 (75.6) | 1.04 (95% CI: 0.87-1.25, p=0.636) | 1.02 (95% CI: 0.85-1.23, p=0.831) |
| *Hypomethylating agent* | no | 597 (42.9) | - | - |
|  | yeas | 796 (57.1) | 0.84 (95% CI: 0.72-0.98, p=0.024) | 0.90 (95% CI: 0.77-1.05, p=0.180) |
| *Total body irradiation* | no | 1206 (86.6) | - |  |
|  | *yes* | 187 (13.4) | 0.91 (95% CI: 0.73-1.14, p=0.424) |  |
| *Anti-thymoglobulines* | no | 512 (36.8) | - |  |
|  | yes | 881 (63.2) | 1.09 (95% CI: 0.93-1.28, p=0.277) |  |
| *Prior treatment* | no | 1166 (83.7) | - |  |
|  | yes | 227 (16.3) | 1.10 (95% CI: 0.90-1.34, p=0.366) |  |
| *Peripheral blood blasts* | 0 | 1058 (76.0) | - | - |
|  | 1 | 335 (24.0) | 2.14 (95% CI: 1.82-2.51, p<0.001) | 2.09 (95% CI: 1.77-2.47, p<0.001) |
| *Marrow blasts* | ≤2 | 235 (56.4) | - |  |
|  | >2 | 182 (43.6) | 0.99 (95% CI: 0.74-1.33, p=0.949) |  |
| *Platelet count (x10^9^/L)* | ≥100 | 192 (13.8) | - |  |
|  | < 100 | 1201 (86.2) | 1.12 (95% CI: 0.89-1.41, p=0.334) |  |
| *Disease stage* | Complete remission | 490 (35.2) | - |  |
|  | Other stages | 903 (64.8) | 1.12 (95% CI: 0.95-1.31, p=0.182) |  |
| *Hemoglobin (g/dl)* | ≥10 | 647 (46.4) | - | - |
|  | <10 | 746 (53.6) | 1.20 (95% CI: 1.03-1.40, p=0.020) | 1.02 (95% CI: 0.87-1.19, p=0.842) |
| *Absolute Neutrophile count (x10^9^/L)* | ≥0.8 | 773 (55.5) | - | - |
|  | <0.8 | 620 (44.5) | 1.19 (95% CI: 1.02-1.38, p=0.027) | 1.15 (95% CI: 0.99-1.34, p=0.073) |
| *T-cell depletion* | no | 452 (32.4) | - |  |
|  | yes | 941 (67.6) | 1.07 (95% CI: 0.91-1.26, p=0.398) |  |

*(Dependent: Surv(dfs.time,dfs), strata = cyt_dg1)*

**Supplementary appendix**

**Table 2s**: Univariate analysis of the association of RIC/MAC regimen and patient’s characteristics

|  |  | MAC  n (%) | RIC  n (%) | OR (95% CI, p) |
| --- | --- | --- | --- | --- |
| *Age (years)* | <60 | 176 (37.8) | 290 (62.2) | - |
|  | >=60 | 164 (17.7) | 763 (82.3) | 2.82 (95% CI: 2.20-3.64, p<0.001) |
| *Sexe* | 1 | 211 (22.9) | 711 (77.1) | - |
|  | 2 | 129 (27.4) | 342 (72.6) | 0.79 (95% CI: 0.61-1.02, p=0.064) |
| *WHO classification* | Others | 73 (23.5) | 238 (76.5) | - |
|  | RAEB-1&2,RA & RCMD | 267 (24.7) | 815 (75.3) | 0.94 (95% CI: 0.69-1.25, p=0.663) |
| *Secondary AML* | saml.no | 304 (24.7) | 929 (75.3) | - |
|  | saml.yes | 36 (22.5) | 124 (77.5) | 1.13 (95% CI: 0.77-1.69, p=0.551) |
| *Cytogentic score* | vgood/good | 227 (24.4) | 705 (75.6) | - |
|  | intermediate | 68 (27.2) | 182 (72.8) | 0.86 (95% CI: 0.63-1.19, p=0.356) |
|  | poor/vpoor | 45 (21.3) | 166 (78.7) | 1.19 (95% CI: 0.83-1.72, p=0.351) |
| *Karnovsky preformance score* | 1 | 255 (26.5) | 709 (73.5) | - |
|  | 2 | 85 (19.8) | 344 (80.2) | 1.46 (95% CI: 1.11-1.93, p=0.008) |
| *IPSSR* | v.low/low | 135 (22.6) | 463 (77.4) | - |
|  | intermediate/high | 158 (25.6) | 459 (74.4) | 0.85 (95% CI: 0.65-1.10, p=0.217) |
|  | v. high | 47 (26.4) | 131 (73.6) | 0.81 (95% CI: 0.56-1.20, p=0.290) |
| *HCT-CI* | 1 | 154 (24.5) | 475 (75.5) | - |
|  | 2 | 84 (22.8) | 285 (77.2) | 1.10 (95% CI: 0.81-1.49, p=0.539) |
|  | 3 | 102 (25.8) | 293 (74.2) | 0.93 (95% CI: 0.70-1.25, p=0.630) |
| *Patient CMV status* | negative | 121 (22.8) | 409 (77.2) | - |
|  | positive | 219 (25.4) | 644 (74.6) | 0.87 (95% CI: 0.67-1.12, p=0.283) |
| *Donor type* | MRD/MUD | 241 (24.3) | 749 (75.7) | - |
|  | Haplo/MMUD | 99 (24.6) | 304 (75.4) | 0.99 (95% CI: 0.76-1.30, p=0.930) |
| *Cell sources* | peripheral blood | 288 (23.0) | 966 (77.0) | - |
|  | others | 52 (37.4) | 87 (62.6) | 0.50 (95% CI: 0.35-0.72, p<0.001) |
| *Hypomethylating agents* | no | 154 (25.8) | 443 (74.2) | - |
|  | yes | 186 (23.4) | 610 (76.6) | 1.14 (95% CI: 0.89-1.46, p=0.296) |
| *Total body irradiation* | *no* | 330 (27.4) | 876 (72.6) | - |
|  | yes | 10 (5.3) | 177 (94.7) | 6.67 (95% CI: 3.67-13.63, p<0.001) |
| *Ant-thymoglobulins* | no | 125 (24.4) | 387 (75.6) | - |
|  | yes | 215 (24.4) | 666 (75.6) | 1.00 (95% CI: 0.78-1.29, p=0.997) |
| *Prior treatment* | no | 258 (22.1) | 908 (77.9) | - |
|  | yes | 82 (36.1) | 145 (63.9) | 0.50 (95% CI: 0.37-0.68, p<0.001) |
| *Peripheral blood blasts* | 0 | 264 (25.0) | 794 (75.0) | - |
|  | >0 | 76 (22.7) | 259 (77.3) | 1.13 (95% CI: 0.85-1.52, p=0.400) |
| *Marow blasts* | <=2 | 127 (21.0) | 477 (79.0) | - |
|  | >2 | 213 (27.0) | 576 (73.0) | 0.72 (95% CI: 0.56-0.92, p=0.010) |
| *Platelet count (x10^9^/L)* | >=100 | 26 (13.5) | 166 (86.5) | - |
|  | < 100 | 314 (26.1) | 887 (73.9) | 0.44 (95% CI: 0.28-0.67, p<0.001) |
| *Disease stage* | CR | 98 (20.0) | 392 (80.0) | - |
|  | Other stages | 242 (26.8) | 661 (73.2) | 0.68 (95% CI: 0.52-0.89, p=0.005) |
| *Hemoglobin (g/dL)* | >=10 | 161 (24.9) | 486 (75.1) | - |
|  | <10 | 179 (24.0) | 567 (76.0) | 1.05 (95% CI: 0.82-1.34, p=0.700) |
| *Absolute neutrophil Count (x10^9^/L)* | >=0.8 | 181 (23.4) | 592 (76.6) | - |
|  | < 0.8 | 159 (25.6) | 461 (74.4) | 0.89 (95% CI: 0.69-1.13, p=0.336) |
| *T-cell depletion* | no | 122 (27.0) | 330 (73.0) | - |
|  | yes | 218 (23.2) | 723 (76.8) | 1.23 (95% CI: 0.95-1.58, p=0.120) |

**Supplementary appendix**

**Table 3s**: Patient’s characteristics after propensity-score matching

| Colonne1 | level | MAC  n (%) | RIC  N (%) | p |
| --- | --- | --- | --- | --- |
| n |  | 333 | 333 |  |
| *Age (%)* | < 60 | 169 (50.8) | 169 (50.8) | 1.000 |
|  | ≥ 60 | 164 (49.2) | 164 (49.2) |  |
| WHO classification (%) | Others | 77 (23.1) | 95 (28.5) | 0.132 |
|  | RA | 256 (76.9) | 238 (71.5) |  |
| Secondary AML(%) | saml.no | 35 (10.5) | 51 (15.3) | 0.083 |
|  | saml.yes | 298 (89.5) | 282 (84.7) |  |
| Cytogenetic score (%) | vgood/good | 223 (67.0) | 200 (60.1) | 0.109 |
|  | interm | 65 (19.5) | 70 (21.0) |  |
|  | poor/vpoor | 45 (13.5) | 63 (18.9) |  |
| IPSSR (%) | very low/low | 132 (39.6) | 116 (34.8) | 0.231 |
|  | intermediate/high | 85 (25.5) | 80 (24.0) |  |
|  | very high | 116 (34.8) | 137 (41.1) |  |
| HCT-CI risk (%) | 1 | 152 (45.6) | 146 (43.8) | 0.697 |
|  | >1 | 181 (54.4) | 187 (56.2) |  |
| CMV status of patient (%) | pat.cmv- | 117 (35.1) | 128 (38.4) | 0.422 |
|  | pat.cmv+ | 216 64.9) | 205 (61.6) |  |
| Donor type (%) | MDR/MUD | 235 (70.6) | 242 (72.7) | 0.606 |
|  | Haplo/MMUD | 98 (29.4) | 91 (27.3) |  |
| Source of cells (%) | 1 | 41 (12.3) | 41 (12.3) | 1.000 |
| Hypomethylating agents (%) | hma.no | 151 (45.3) | 139 (41.7) | 0.390 |
|  | hma.yes | 182 (54.7) | 194 (58.3) |  |
| Prior-treatment (%) | no | 77 (25.6) | 73 (24.8) | 0.907 |
|  | yes | 224 (74.4) | 221 (75.2) |  |
| Hemoglobin (%) | ≥ 10 g/dl | 59 (17.7) | 50 (15.0) | 0.402 |
|  | < 10 g/dl | 274 (82.3) | 283 (85.0) |  |
| Peripheral blood blasts (%) | 0 | 283 (85.0) | 281 (84.4) | 0.914 |
|  | 1 | 50 (15.0) | 52 (15.6) |  |
| Marrow blasts (%) | =<1 | 84 (25.2) | 86 (25.8) | 0.929 |
|  | >1 | 249 (74.8) | 247 (74.2) |  |
| Platelet (x 10^9^/L(%) | ≥ 100 | 116 (34.8) | 143 (42.9) | 0.039 |
|  | < 100 | 217 (65.2) | 190 (57.1) |  |
| Absolute Neutrophile Count (x 10^9^/L) (%) | ≥ 0.8 | 271 (81.4) | 263 (79.0) | 0.496 |
|  | < 0.8 | 62 (18.6) | 70 (21.0) |  |
| Disease stage (%) | Complete remission | 96 (28.8) | 96 (28.8) | 1.000 |
|  | Other stages | 237 (71.2) | 237 (71.2) |  |

**Figure 1s**: Kaplan-Meier curves and Log-rank of the association of RIC/MAC conditioning regimens on Overall survival and Non-relapse mortality outcomes according to subgroups which could benefit from either RIC or MAC.

| 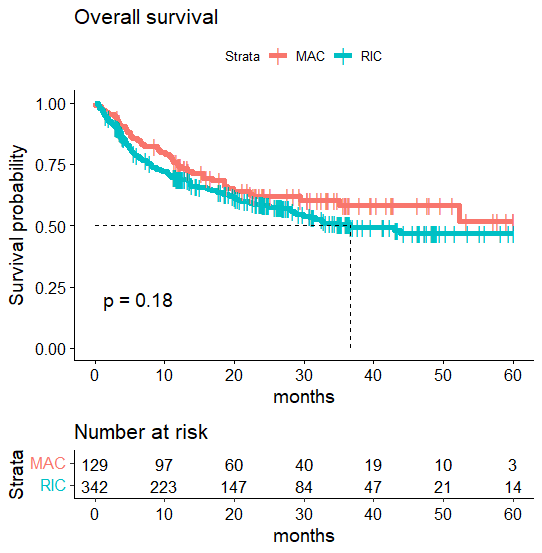 | 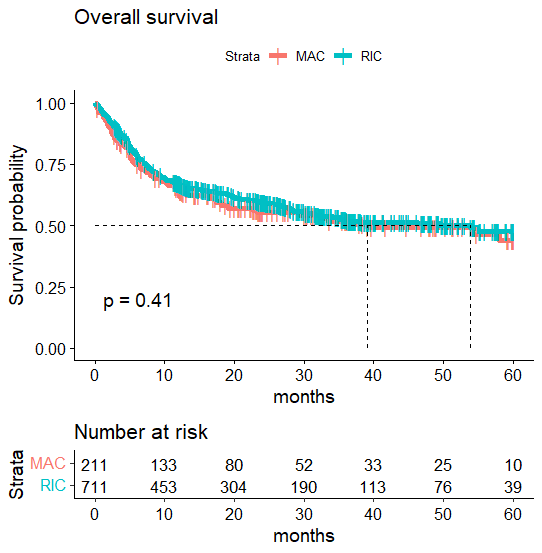 |
| --- | --- |
| Overall survival curves of RIC/MAC regimen in the MAC regimen recommended subgroup | Overall survival curves of RIC/MAC regimen in the RIC regimen recommended subgroup |

| 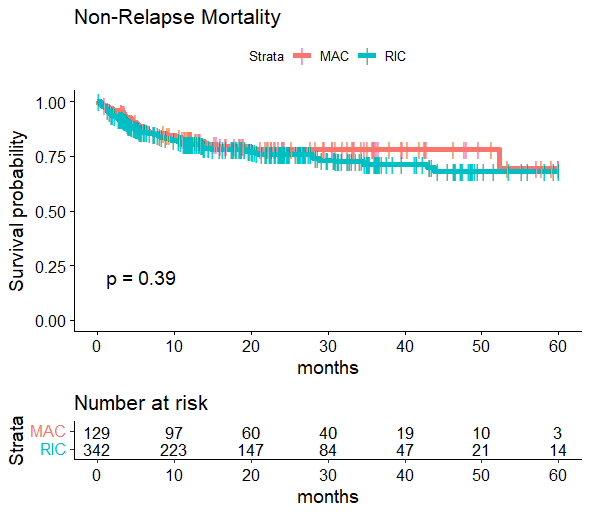 | 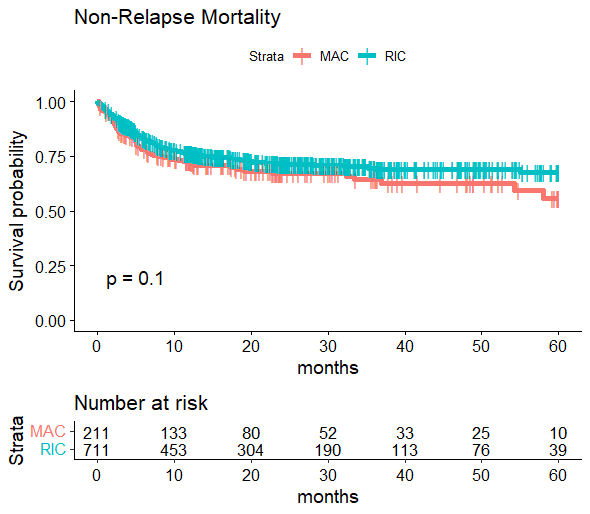 |
| --- | --- |
| Non-relapse mortality curves of RIC/MAC regimen in the MAC regimen recommended subgroup | Non-relapse mortality curves of RIC/MAC regimen in the RIC regimen recommended subgroup |

**Figure 2s**: Kaplan-Meier curves and Log-rank of the association of RIC/MAC conditioning regimens on Overall survival, Event-free survival, and cumulative hazard curves for “Relapse” and “Non-relapse mortality” outcomes after removing transformed into AML patients from the analysis.

| 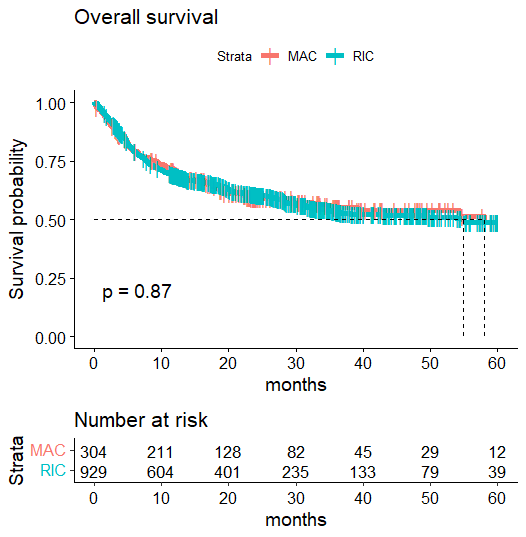 | 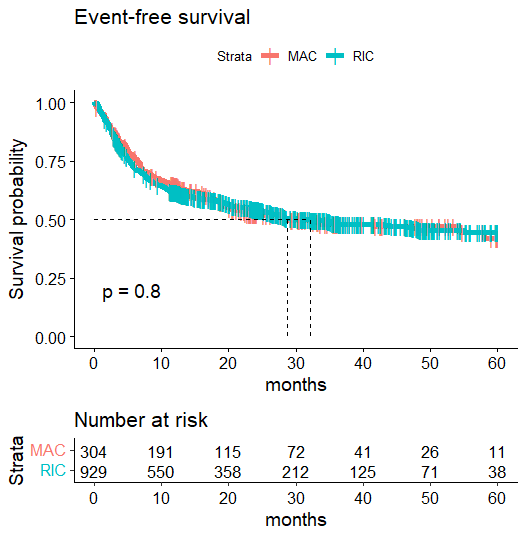 |
| --- | --- |
| 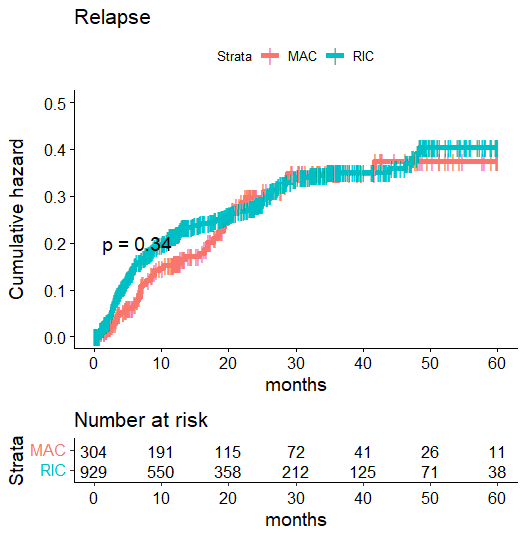 | 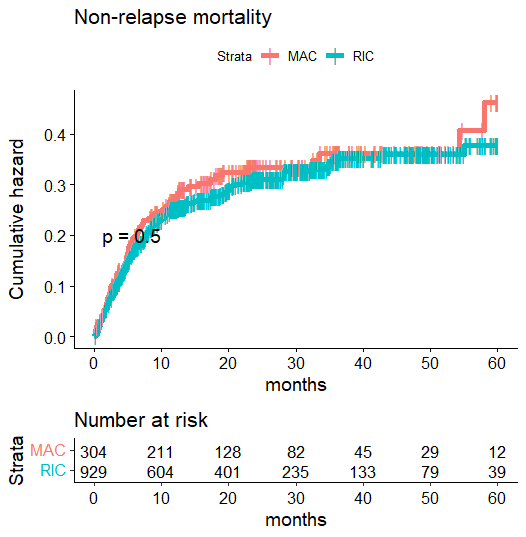 |
